# Filling In the Gaps: Ethylene Glycol Poisoning Presenting With Isolated Lactate and Osmolar Gaps

**DOI:** 10.7759/cureus.54749

**Published:** 2024-02-23

**Authors:** Caden Quintanilla, Justin Panthappattu, Davood Hosseini, Karan Omidvari

**Affiliations:** 1 Internal Medicine, Hackensack University Medical Center, Hackensack, USA; 2 Medicine, Hackensack University Medical Center, Hackensack, USA

**Keywords:** toxicity, osmolar concentration, lactic acid, toxicology, etethylene glycol

## Abstract

Ethylene glycol (EG) is an organic compound used in antifreeze. In 2020 alone, there were 5,277 EG exposures, with only 617 reported as intentional ingestions. Therefore, encountering EG toxicity is rare; however, it is essential to identify it promptly based on a focused history, exam, and rapid identification of commonly associated EG-induced metabolic derangements. If the diagnosis is not made within 12 hours of ingestion or exposure, severe morbidity and mortality can occur. Previous reports of EG poisoning have occurred in the setting of a lactate gap (LG) and osmolar gap (OG); however, they also had commonly associated findings of EG toxicity such as high anion gap acidosis (HAGMA), acute kidney injury (AKI), hypocalcemia, calcium oxalate stones, and suggestive histories of EG ingestion. We present a case of a 57-year-old male who presented from home for slurred speech and gait imbalance. He was intubated for airway protection due to obtundation. Labs only revealed the presence of both LG and OG, non-anion gap acidosis (NAGMA), and an EG level of 112 mg/dL three days after admission. Hemodialysis (HD) was initiated solely based on these findings within eight hours of admission, and he was subsequently able to be extubated without developing an acute or chronic cardio-pulmonary or renal injury. The patient’s partner reported to the care team that they found multiple empty bottles of rum and whisky, an empty anti-freeze bottle, and a Sprite bottle with a light blue substance that was nearly empty in their basement. After extubation, the patient admitted to ingesting the antifreeze with the intention of self-harm. He recovered without complication and was transferred to the inpatient psychiatric unit to manage his depression and suicidality further. The early diagnosis and treatment of EG poisoning is critical to prevent severe morbidity and mortality occurring only 12 hours after ingestion. Therefore, reliance on prompt recognition of common laboratory findings, understanding of EG toxicity-specific signs and symptoms, and awareness of other rapid diagnostic tools for EG are essential in clinching the diagnosis. This case highlights the potential atypical presentations of EG toxicity, helpful diagnostic strategies, and the importance of avoiding anchoring bias when commonly associated disease processes are absent.

## Introduction

Ethylene glycol (EG) is a colorless, sweet-tasting organic compound used in antifreeze, pesticides, and industrial solvents for paints and plastics [1]. In 2020 alone, 5,277 EG exposures were recorded, with only 617 reported as intentional ingestions [2]. With an estimated 131 million ED visits annually in the United States [3], the likelihood of encountering an intentional EG ingestion is approximately 1 in 200,000. Therefore, the odds of encountering an intentional ingestion are exceptionally low. Still, if it is not diagnosed within twelve hours from the time of ingestion, there are significant increases in morbidity and mortality. This scenario can be challenging to clinicians as it requires rapid identification of a rarely encountered toxicologic process. Since the toxidrome of EG intoxication is seldom encountered, and outcomes are determined by rapid diagnosis and treatment, clinicians must become familiar with the typical and atypical presentations of EG toxidrome. Diagnosis and treatment of EG intoxication hinge on a robust suggestive history, prompt identification of common metabolic derangements, and emergent initiation of hemodialysis (HD). The clinical presentation of EG intoxication most commonly presents with signs and symptoms of slurred speech, ataxia, and rapid obtundation. The common metabolic laboratory findings are that of high anion gap acidosis (HAGMA), acute kidney injury (AKI), hypocalcemia, calcium oxalate stones, and occasionally lactate gaps (LG) and osmolar gaps (OG). However, one of the rarest presentations reported in the literature is a normal anion gap metabolic acidosis (NAGMA) [4]. Previous cases of EG intoxication have reported LG and OG; however, they also had concomitant HAGMA, AKI, and hypocalcemia with histories suggestive of EG ingestion [5,6]. We present a case of a 57-year-old male who presented with slurred speech and gait imbalance and was found to have intentional EG poisoning with NAGMA, LG, and OG.

## Case presentation

A 57-year-old otherwise healthy male with a medical history of tobacco use was brought in to evaluate slurred speech and gait imbalance at 8:00 am. He had reportedly experienced acute onset voice hoarseness and slurred speech before becoming unresponsive around 6:00 am earlier that same morning. His girlfriend stated that he had no current or past history of alcohol or substance abuse, depression, suicidal ideation, recent surgeries, recent new medications or medication changes, or any other significant medical history besides tobacco use. En route to the emergency department (ED), paramedics noted that he was only responsive to painful stimuli. He received naloxone, but it did not improve his condition. At presentation in the ED, he was noted to be hypothermic to 34.5 ℃, bradycardic to 51 beats per minute, and normotensive. Due to obtundation, the patient was intubated for airway protection. The physical exam was unremarkable. Computed tomography (CT) of the head, CT head angiogram, and CT head perfusion scan were unremarkable. Chest x-ray was without acute cardio-pulmonary disease. His initial complete blood count and chemistry were normal, including urine analysis, urine drug screen, and a negative blood alcohol level.

After evaluation by the intensive care unit (ICU) consult team, it was discovered the patient on arterial blood gas (ABG) with lactate had a pH of 7.20 and lactate of 9.75 mmol/L; however, his serum lactate resulted as only 1.5 mmol/L. His bedside point-of-care ultrasound (POCUS) revealed normal RV and LV function without any notable valvular abnormalities or pericardial effusion (for the complete initial workup, please see Tables 1, 2). When the patient was handed off to the primary ICU care team, the first assessment was that the patient had encephalopathy of unclear etiology, most likely seizures, as well as lactic acidosis with an abnormal discrepancy between the serum and ABG lactate result. The plan was to repeat the serum and ABG with lactate to see if they were artifactual, as a rapidly rising lactate level would be concerning for acute bowel perforation or ischemia. The repeat ABG with lactate was completed at 12:30 pm (six hours after his reported mental status change), showing a pH of 7.179 and a rise in lactate to 12.51 mmol/L, but the repeat serum lactate was normal at 1.1 mmol/L. At this point, the suspicion of toxic alcohol ingestion was high despite the absence of other typical metabolic findings associated with their ingestion. A serum osmolality of 327 mOsm/kg was obtained, with a calculated elevated Osm gap of 39 mOsm/kg. The ICU team re-interviewed the patient's partner about alcohol use, depression, or any suicidality, which she adamantly denied. A toxicology consult was placed, and the recommendation was made to empirically treat for unknown volatile alcohol ingestion with fomepizole 15mg/kg, thiamine 100mg IV once daily for three days, and pyridoxine 100mg IV twice daily for three days, and proceed with emergent HD for four hours and to trend the osmolality, chemistry, and pH every four hours for 12 hours to determine further need for HD. A serum toxic alcohol panel was sent, but it would take approximately two to three days to result, which included EG, methanol (MET), and isopropanol (ISO) levels. Based on the assessment up to this point and after a detailed discussion with the patient’s significant other, the decision was made to initiate HD. HD had been started within eight hours of presentation to the hospital. 

**Table 1:** Basic metabolic panel and arterial blood gases before and after dialysis. Blood Urea Nitrogen is abbreviated as “BUN.” Abnormal results denoted by “*”

	Pre-Dialysis	Post-Dialysis	Reference Range
Basic Metabolic Panel			
Calcium (mg/dL)	8.6	8.1*	8.4-10.2
Bicarbonate (mmol/L)	22	24	22 - 29
BUN (mg/dL)	14	8	7 - 18.7
Creatinine (mg/dL)	0.87	0.98	0.3 - 1.50
Anion Gap	10	5	<12
Arterial Blood Gas			
pH	7.2*	7.3*	7.35 - 7.45
pCO2 (mmHg)	34.3*	43.2	35 - 45
pO2 (mmHg)	191.5*	103.8*	75 - 100
Lactate (mmol/L)	9.75*	2.17	0.5-2.2

**Table 2:** Urinalysis and other miscellaneous labs before and after hemodialysis Abnormal results denoted by “*”

	Pre-Dialysis	Post-Dialysis	Reference Range
Urinalysis			
Glucose (mg/dL)	Negative	Negative	Negative
Ketones (mg/dL)	Negative	15*	Negative
Protein (mg/dL)	Negative	100*	Negative
Red Blood Cells (mg/dL)	Negative	>50*	Negative
Calcium Oxalate Stones	Negative	Present	Negative
Miscellaneous			
Serum Lactate (mmol/L)	1.5	Not obtained	0.5-2.2
Ethylene Glycol Level (mg/dL)	112*	Not obtained	<20
Osmolality Gap (mOsm/kg)	39*	4	-14 to +10

Complete post-HD labs can be viewed in Table 1 but were most significant for a repeat UA with calcium oxalate crystals, the presence of protein and greater than 50 red blood cells per high power field, a serum Osm gap of 4 mOsm/kg, and the ABG with lactate with a pH of 7.30 and lactate of 2.17 mmol/L. Based on this improvement, after only one session of HD, the decision was made with nephrology and toxicology to remove the Shiley catheter, with no further sessions of HD required. The patient continued to have appropriate urine output without evidence of kidney injury on subsequent chemistries. The 24-hour video electroencephalogram was unremarkable for seizure activity and consistent with diffuse cerebral slowing. On day 3 of hospitalization (D3H), the serum EG level returned positive at 112mg/dL, negative for MET or ISO. The patient’s partner reported to the care team that they found multiple empty bottles of rum and whisky, an anti-freeze bottle, and a Sprite bottle with a light blue substance that was nearly empty in their basement. An MRI brain completed on D4H showed no abnormalities. On D5H, the patient was extubated and admitted to intentionally ingesting antifreeze. Increased secretions and low-grade temperatures complicated his ICU course. He was found on a repeat chest x-ray to have a new right lower lobe infiltrate, which was treated with antibiotics. He also had an episode of visual hallucinations that resolved spontaneously. The patient was then transferred from the ICU to the inpatient voluntary psychiatric ward to manage depression and suicidality further. He is currently recovering well and being managed on an outpatient basis for his continued psychiatric needs.

## Discussion

**Figure 1:**
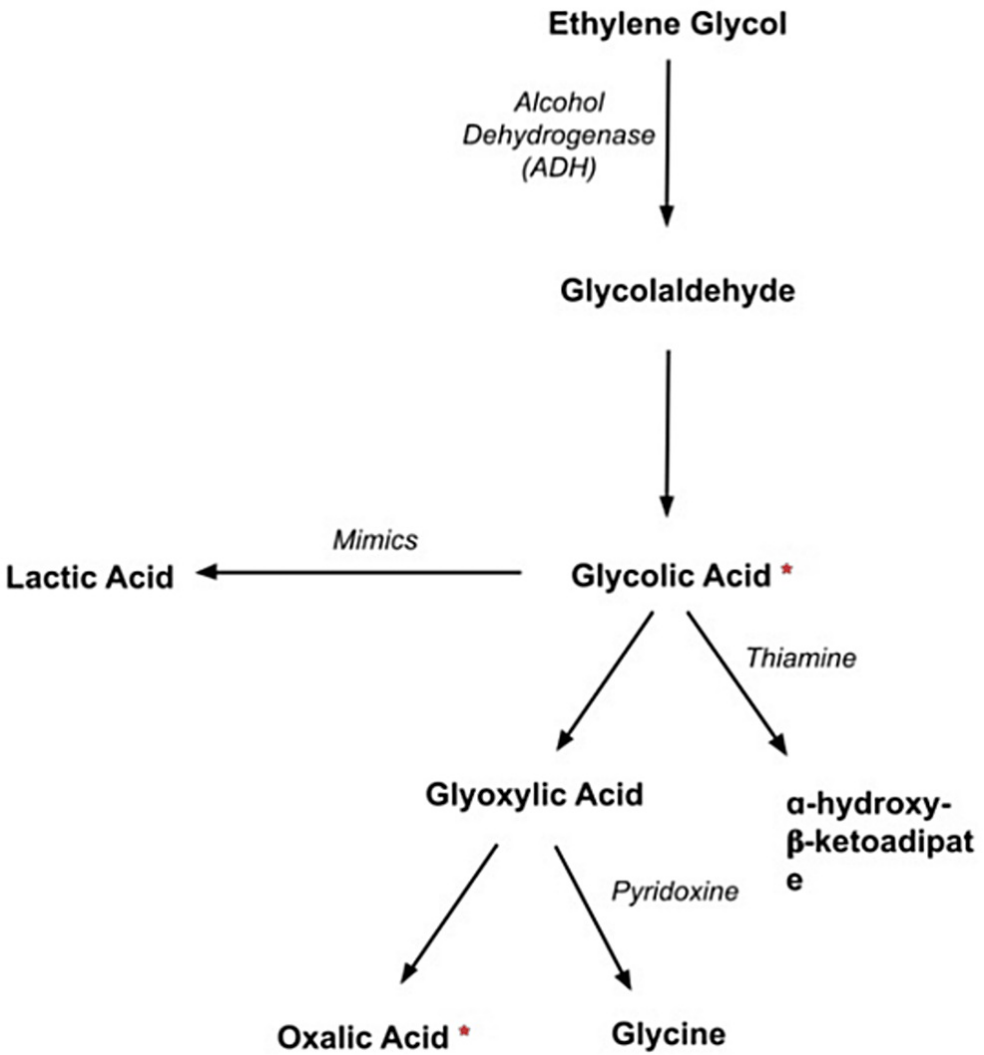
EG metabolism is initiated by the enzyme ADH, which produces the toxic metabolites of glycolic acid and oxalic acid denoted with “*." Thiamine and pyridoxine administration increase the shunting of toxic metabolites to non-toxic metabolites. Increased EG metabolism produces more glycolic acid, which is interpreted by point-of-care ABG analyzers as falsely elevated serum lactate levels, creating the lactate gap when compared to serum assays

EG intoxication classically manifests with acute changes in mental status, ataxia, HAGMA, AKI, suggestive histories, and hypocalcemia complicated by calcium oxalate urinary stones. In the literature, the rarest presentations of EG toxicity have been that of a NAGMA; however, even in these cases, other suggestive factors were present [4]. Clinching the diagnosis requires a high index of suspicion. This case represents an atypical presentation of a rarely encountered disease process based on multiple factors. To start, the collateral history obtained from the patient's significant other was helpful but also detrimental to obtaining the correct diagnosis as she reported his abnormal wide-based gait, slurred speech, and acutely becoming unresponsive, but they also adamantly denied the patient was suicidal or had any alcohol or substance abuse. The initial differential was most concerning for an acute ischemic or hemorrhagic stroke, given the abrupt onset of symptoms and severity of encephalopathy, which was immediately ruled out with CT imaging and later confirmed on an MRI brain. Sepsis was also considered in this case, given the initial hypothermia; however, he was without any apparent source of infection. It was not until three days after intubation that the patient had increased secretions and low-grade temperatures, which prompted a new chest x-ray that showed a new infiltrate at the right base that was not present on admission. His blood cultures were negative. The other primary differential would be non-convulsive status epilepticus, overdose, or toxic ingestions, such as opioid-induced narcosis. Of note, he did not improve after naloxone administration, and his exam was without classic toxidromic findings, such as lead-pipe rigidity, diaphoresis, or pupillary abnormalities. He also had a negative 24-hour video electroencephalogram. Metabolically, his initial chemistry and ABG were consistent with a NAGMA. The primary driver of the continued urgent diagnostic evaluation was the unexplained rising lactate observed on ABG but normal levels on peripheral serum lactate draw. This phenomenon is known as the LG, which raises the suspicion of toxic alcohol ingestion. The LG is created by the way lactate is measured via modern assays. Point-of-care ABG analyzers use hydrogen peroxide (H2O2) as a surrogate marker for lactate concentration in the blood. EG is non-toxic; however, it is rapidly metabolized by the enzyme alcohol dehydrogenase (ADH) into the toxic metabolites of glycolic acid (GA) and oxalic acid (OA) (Figure 1). Most point-of-care ABG analyzers utilize the enzyme L-lactate oxidase, which oxygenates lactate and GA, producing H2O2. Therefore, the analyzers do not discriminate between the two structures and still produce high amounts of H2O2 (Figure 1). Thus, the analyzers will report falsely elevated lactate levels, while the serum measurement reveals normal lactate concentrations. However, it is essential to note that there can be true lactic acidosis in EG toxicity and a normal OG [5,6]. The patient did have a classic EG intoxication presentation of suspected ataxia, slurred speech, and severe encephalopathy; however, he was without the commonly associated metabolic findings of HAGMA, AKI, or hypocalcemia. Therefore, the care team had to rely on the presence of the LG and the OG as the sole suggestive metabolic factors of EG ingestion among the already broad differential. It was prudent of the care team not to anchor on suspicion of acute abdominal perforation or non-convulsive status epilepticus in this case. 

The pathophysiology of EG toxicity can be thought of as developing in three stages based primarily on time from ingestion and whether or not co-ingestion with ethanol occurred. Stage one occurs from 3-12 hours after ingestion and is primarily characterized by the central nervous system (CNS) depression manifesting as severe inebriation without the scent of ethanol in the breath, ataxia, coma, and metabolic derangements, principally that of an anion gap metabolic acidosis. The acidosis, when present, is usually severe, with a pH of less than 6.9 and bicarbonate levels of less than 5 mEq/L due to the rapid production of GA in the blood. Rapid obtundation after EG ingestion is because EG is a much more potent depressant than the more commonly encountered ethanol due to faster gastric and small intestinal absorption [7]. Stage two occurs 12-48 hours post-digestion and is where most deaths occur. It is defined by noncardiogenic acute respiratory distress syndrome, congestive heart failure, severe hypocalcemia, renal failure, and severe metabolic acidosis [7]. Stage three is 48 hours and beyond and is predominantly manifested by renal failure and the development of bone marrow suppression characterized by pancytopenia [7]. GA drives the cardiopulmonary manifestations of toxicity by interrupting oxidative phosphorylation and cellular respiration. OA leads to renal failure by the precipitation of calcium oxalate stones, leading to direct tubular necrosis, primarily of the proximal tubules, which can lead to glucosuria, proteinuria, and hematuria [7]. It is important to note that these stages can be prolonged based on whether or not congestion with ethanol occurs, which will delay the metabolism of EG and, therefore, delay the onset of end-organ damage. Interestingly, intravenous and oral ethanol used to be part of the mainstay of treatment for suspected EG toxicity due to this effect [7].

In this case, our patient’s symptoms fit stage one of ingestion (within 12 hours of the ingestion) as he primarily had CNS depressant effects of rapid obtundation and ataxia, developed a NAGMA, and elevated serum osmolality. His initial UA was without calcium oxalate stones or evidence of tubular injury, also lending evidence that the time of his ingestion had to have been near the time when he developed slurred speech and encephalopathy. However, after one session of HD, the repeat UA was significant for the presence of calcium oxalate stones, mild proteinuria, and hematuria. This indicated at least some direct tubular toxicity due to calcium oxalate stone precipitation in the proximal tubules but, thankfully, was without the progression to clinically significant AKI as evidenced by subsequent chemistries. It is likely that none of the classic findings, such as HAGMA, AKI, or hypocalcemia, were seen based on how early the patient presented to the ED from the time of ingestion and the prompt initiation of treatment. The mainstay of therapy for EG toxicity consists of ADH blockade with fomepizole, clearance of EG and its toxic metabolites via HD, and high-dose thiamine and pyridoxine to drive the metabolism of GA and glyoxylic acid into harmless products of ɑ-hydroxy-𝛽-ketoadipate and glycine respectively (Figure 1) [7]. Remember that magnesium is a co-factor for thiamine in this alternate degradation pathway and should be repleted aggressively when hypomagnesemia develops. HD should be initiated when the EG level is greater than 50 mg/dL, the presence of severe acidosis, severe electrolyte abnormalities not responsive to conventional therapy, or any observed kidney injury. Other studies suggest that HD should be continued until the EG level is less than 1.6mmol/L. However, practically, this is not always feasible, given the delays in obtaining EG levels [5]. In severe acidosis, aggressive intravenous bicarbonate therapy is indicated as a bridge to initiation of HD. Calcium oxalate stones form more readily in an acidic environment, and therefore, alkalinization of the urine may help reduce precipitation with a therapeutic goal of a urine pH of 7; however, this has never been validated in clinical trials [5,7]. If there is the presence of hypocalcemia, clinical observation for the presence of tetany should occur, with the supplementation of calcium as necessary. When HD is started, metabolic corrections should be expected to occur immediately in post-HD labs, including the OG and LG, as was observed in this case. To ensure the appropriate corrections occur, trending the serum pH, OG and anion gap is essential. Further sessions of HD may be required as EG redistributes in the tissues between HD sessions, which can lead to further metabolism of EG into its toxic metabolites [7]. Patients who survive the initial stages of EG toxicity mostly have renal recovery and do not require long-term HD or renal transplant [7]. This patient has had continued outpatient psychiatric management of his depression and has not had any long-term cardio-pulmonary or renal impairment as a result of his EG ingestion.

## Conclusions

Early diagnosis and treatment are critical to ensure the most advantageous morbidity and mortality outcomes in EG toxicity. This can be extremely challenging since rapid laboratory assays of EG, MET, and isopropyl (ISO) levels are not readily available and may take up to 48 hours to result. Therefore, reliance on prompt recognition of common laboratory findings, understanding of EG toxicity-specific signs and symptoms, and awareness of other rapid diagnostic tools for EG are essential in clinching the diagnosis. This case highlights the potential atypical presentations of EG toxicity, helpful diagnostic strategies, and the importance of avoiding anchoring bias when commonly associated disease processes are absent.
